# The Role of 3 Tesla MRA in the Detection of Intracranial Aneurysms

**DOI:** 10.1155/2012/792834

**Published:** 2012-01-16

**Authors:** Eftychia Z. Kapsalaki, Christos D. Rountas, Kostas N. Fountas

**Affiliations:** ^1^Department of Diagnostic Radiology, University Hospital of Larissa, Faculty of Medicine, University of Thessaly, 41110 Larissa, Greece; ^2^Department of Neurosurgery, University Hospital of Larissa, Faculty of Medicine, University of Thessaly, Biopolis, 41110 Larissa, Greece; ^3^Institute of Biomedical Research & Technology (BIOMED), Center for Research and Technology-Thessaly (CERETETH), 41222 Larissa, Greece

## Abstract

Intracranial aneurysms constitute a common pathological entity, affecting approximately 1–8% of the general population. Their early detection is essential for their prompt treatment. Digital subtraction angiography is considered the imaging method of choice. However, other noninvasive methodologies such as CTA and MRA have been employed in the investigation of patients with suspected aneurysms. MRA is a noninvasive angiographic modality requiring no radiation exposure. However, its sensitivity and diagnostic accuracy were initially inadequate. Several MRA techniques have been developed for overcoming all these drawbacks and for improving its sensitivity. 3D TOF MRA and contrast-enhanced MRA are the most commonly employed techniques. The introduction of 3 T magnetic field further increased MRA's sensitivity, allowing detection of aneurysms smaller than 3 mm. The development of newer MRA techniques may provide valuable information regarding the flow characteristics of an aneurysm. Meticulous knowledge of MRA's limitations and pitfalls is of paramount importance for avoiding any erroneous interpretation of its findings.

## 1. Introduction

It is well known that intracranial aneurysms are identified in 1–8% of the general population [1]. They represent the most common cause of nontraumatic subarachnoid hemorrhage. Unruptured aneurysms do not usually cause symptoms, unless they rupture or when they compress adjacent neural structures causing focal neurological deficits. Digital subtraction angiography (DSA) is still considered the gold standard among the currently used imaging methods for the diagnosis of an intracranial aneurysm. However, DSA is an invasive diagnostic modality, with very low but occasionally troublesome morbidity [2]. Other noninvasive imaging modalities have been developed for imaging the intracranial vessels and detecting aneurysms or other vascular pathology. Computed tomographic angiography (CTA) and magnetic resonance angiography (MRA) are routinely utilized nowadays in clinical practice. These noninvasive imaging methods have undergone significant advances in image quality becoming thus more and more sensitive and accurate. They can precisely depict not only the presence of an aneurysm but they can also provide valuable information regarding the size, the shape, and the hemodynamic flow characteristics of an intracranial aneurysm.

Patients presenting with subarachnoid hemorrhage (SAH) are initially investigated with CTA. This modality is readily available and provides quite accurate information regarding the cause of SAH in a timely fashion. The use of intravenous contrast media is necessary for the identification of blood vessels and provides accurate information regarding the location, the size, and the shape of an aneurysm, as well as the presence of multiple aneurysms. It has, however, the disadvantage of radiation exposure and the possibility of an allergic reaction to the iodinated contrast agents [3, 4].

Likewise, asymptomatic patients that need to be investigated for the presence of an intracranial aneurysm should undergo a minimally invasive diagnostic procedure, as CTA or MRA. A lot of controversy still exists regarding the treatment of incidentally identified intracranial aneurysms. According to the International Study of Unruptured Intracranial Aneurysms Investigators (ISUIAI), the rupture rate of small aneurysms (<1 cm) is 0.05%/year, while that of aneurysms larger than 1 cm or aneurysms that have previously ruptured is 0.5%/year [5]. The optimal management of an unruptured aneurysm remains ill-defined, and definitely the therapeutic decision depends on several parameters. Furthermore, the necessity of screening the general population for an intracranial aneurysm is disputable. Specific patient populations present an increased risk for intracranial aneurysms. These are patients with polycystic kidney disease, Marfan syndrome, coarctation of the aorta, fibromuscular dysplasia, family history of saccular aneurysm, and Ehlers-Danlos syndrome. In such patients, a minimally invasive, sensitive, and highly accurate method needs to be available for their investigation.

In our present study, we examine the role of 3 T MRA in the detection and treatment decision algorithm of intracranial aneurysms. We present a brief historical overview of MRA, the currently used techniques, their pitfalls, and the MRA's clinical significance by systematically reviewing the pertinent literature.

## 2. Historical Overview

Digital subtraction angiography (DSA) is considered to be the gold standard for the diagnosis of intracranial aneurysms. This method provides detailed information regarding the presence, the anatomic location, and the morphology of an aneurysm [6, 7]. It demonstrates the relationship of an aneurysm with its parent vessel as well as the adjacent vessels and also provides important aneurysmal flow dynamic information. In a routine DSA study, a large amount of contrast material is required. Moreover, in order to identify the aneurysm and its relationship with the parent vessel, very frequently multiple rotational and oblique views are obtained, thus increasing both the injected contrast as well as the time of the examination. A specific protocol has to be followed according to the published guidelines in order to minimize the amount of radiation exposure for the patients and for the involved medical personnel [8, 9]. Furthermore, the introduction of flat panel detector (FPD) technology has greatly reduced radiation doses, while it has improved image quality. The use of FPD technology provides high spatial resolution, wide dynamic range, and real time imaging capabilities [10]. These FPD features allow the safer acquisition of more rotational angiographic data and consequently the creation of high-resolution 3D DSA images. Moreover, the introduction of 3D DSA provided a more precise diagnostic tool compared to 2D DSA and has become a tool of paramount importance for the endovascular treatment of an aneurysm [11]. However, DSA is an invasive method and presents a very low (<1%) but not insignificant risk of neurological complications [12, 13]. Furthermore, it has been associated with other minor complications as hematoma formation at the puncture site, pseudoaneurysm, and more rarely the development of an arteriovenous fistula at the puncture site. Finally, allergic reaction or nephrotoxicity due to the iodinated contrast agents may be rare but serious complications [3, 4]. It needs to be mentioned that even though DSA is still considered the gold standard for imaging intracranial aneurysms, in patients presenting with SAH, CTA is generally accepted as the initial method of evaluation.

Computed tomography (CT) is the initial method of choice in evaluating patients presenting with possible SAH. Computed tomographic angiography may also be performed at the same time and may provide valuable information regarding the cause of SAH. In the early era of single slice CT scanners, CTA was able to identify only intracranial aneurysms sized 5 mm or larger. Initially, only a small field of view could be included and appreciated with CTA, since scanning was not fast enough to cover larger areas. Thus, CTA was not adequate for evaluating patients with SAH. The evolution of CT, the introduction of multichannel CT scanners (4, 8, 16, 32, and 64), and the use of cone beam CT improved the obtained resolution and diagnostic accuracy, thus making CTA's sensitivity comparable to that of DSA [14–16]. The CTA technique requires injection of iodinated contrast media (iodine concentration: 320–400 mg/mL) via an antecubital vein at a flow rate of 3 to 5 mL/s, up to a total of 60 to 100 mL [17]. The covered area extends from C1 vertebra to the top of the head with a slice thickness of 0.6 to 1.25 mm and a reconstruction interval of 0.4 to 0.6 mm. Images may be reconstructed in 2D maximum intensity projection (MIP) or 3D volume rendering (VR). The sensitivity of CTA for 4- and 16-channel scanners is particularly high, depending on the size and the anatomic location of the aneurysm, but has significantly improved with the introduction of 64-channel scanners. Continuing experience with this modality has significantly increased its sensitivity and specificity, which are reported to be as high as 90%, but is dependent on the size of the aneurysm [18–22]. In aneurysms larger than 4 mm, it has been reported that CTA sensitivity was as high as 95%. White et al., in a meta-analysis including studies published from 1988 to 1998, found a global CTA sensitivity of 89% strongly dependent on aneurysmal size, ranging from 61% for aneurysms smaller than 4 mm to 96% for aneurysms larger than 4 mm [18]. The sensitivity of CTA is also dependent on the anatomic location of the aneurysm. More specifically, Villablanca et al. found that CTA has a sensitivity of >90% for aneurysms of the middle cerebral artery, regardless the size of the aneurysm [23, 24]. However, several studies report that CTA may not clearly identify small aneurysms in the area of the carotid siphon, making necessary investigation with other imaging modalities [25–27].

Magnetic resonance angiography is considered to be the preferred screening diagnostic method in asymptomatic patients with an increased possibility of harboring an intracranial aneurysm. It is also considered the preferred imaging method of following up unruptured intracranial aneurysms, since it is a noninvasive method, with no radiation exposure [28]. MRA may determine the presence of one or multiple aneurysms. It may also identify the characteristics of an aneurysm such as its size, its location, the presence and the stereotactic configuration of its neck, and the wall texture of the aneurysmal dome. Furthermore, with the recent introduction of high-field MRI units and the evolution of MR imaging techniques, MRA may identify aneurysms as small as 3 mm, when the optimal protocol is employed [29–31]. White et al. in a systematic review study comparing MRA with DSA showed that MRA has an accuracy of 90%, a sensitivity of 87%, a specificity of 95%, a positive predictive value of 97%, and a negative predictive value of 77% per aneurysm [18].

Thus, MRA has an overall sensitivity of about 93%–97% in detecting aneurysms larger than 3 mm and about 85%–93% in detecting aneurysms smaller than 3 mm, with the application of special techniques at 3 T. The MRA acquisition requires the patient's cooperation, and it lasts approximately 3–6 min. Thus, MRA is not the preferred choice of investigating critically ill patients with SAH in the acute setting. MRA has undergone significant improvements in the last years, with the advancement of MR systems, the introduction of high magnetic fields (3 T) in the routine clinical practice, and the progression of computer software programs.

## 3. MRA Techniques

The techniques used to produce angiographic images with MRI are phase contrast (PC), time of flight (TOF), and contrast-enhanced MRA (CE-MRA).

### 3.1. Phase-Contrast MRA

Phase-contrast (PC) technique acquires two paired data acquisitions with opposite bipolar flow-encoding gradient pulses, resulting in images with vascular signal approximately proportional to the velocity-induced phase shifts [31–33]. As with the other phase-sensitive techniques, the surrounding stationary tissue has identical signal on both acquisitions and thus is subtracted out. Subsequently, only blood vessels are depicted and can be clearly visualized and identified. During PC techniques, the faster the motion of moving cells (blood) is, the larger the signal will be. Phase-contrast images detect motion in one predefined direction, thus permitting only arteries or veins to be identified. Phase-contrast techniques also provide information regarding the velocity of the moving cells [32]. Phase-contrast imaging may be implemented with 2D or 3D acquisition [32–35]. Both 2D and 3D PC-MRA are performed using a thick slab containing the vessels to be imaged. The velocity and the direction of the blood flow need to be preselected, applying a saturation pulse at the periphery of the field of view (FOV). Data may be postprocessed for better identification of blood vessels. Phase-contrast MRA requires a long acquisition time, and thus short TR should be used to reduce the scan time.

### 3.2. Time-of-Flight MRA

Time-of-flight (TOF) MRA sequences provide optimal vascular contrast [36, 37]. Dixon et al. initially used a method that selectively targeted common carotid artery inflow at the carotid bifurcation, with suppression of the stationary tissue using low amplitude gradient pulses [37]. Nishimura et al. used a single slice thick slab at the carotid bifurcation, generating vascular contrast by two acquisitions; one at the carotid bifurcation and one below the carotid bifurcation [38]. The MRA images were produced by subtracting these two acquisitions. Only the vessel signal was identified with this technique.

In 2D TOF technique, images are obtained in the axial plane, perpendicular to the direction of the blood vessels. A saturation band, eliminating venous flow, is placed at the upper edge of the selected slab. This technique provides excellent background suppression and has very good sensitivity to slow flow [39–41]. Keller et al. have enhanced the 2D TOF technique, using a maximal intensity projection (MIP) postprocessing of the acquired data, producing thus better identification of the blood vessels [42]. In 3D TOF technique, images are produced by applying a 3D volume (slab) oriented perpendicular to the direction of flow, producing enhancement of flow, affecting only the spins included in the acquired slab [43–45]. This is attributed to the application of optimal TR and the appropriate flip angle. Multiple overlapping slabs may be used to cover larger regions, which, however, increase the total scan time. Magnetization transfer pulses in combination with fat saturation may be utilized during a 3D TOF MRA study for reducing signal from the surrounding stationary tissues, thus providing improved resolution of the intracranial vessels [43, 44, 46].

### 3.3. Contrast-Enhanced MRA (CE-MRA)

Contrast-enhanced MRA (CE-MRA) techniques are the most recently developed. They are easier to be interpreted and are less susceptible to artifacts, compared to the PC and the TOF techniques [47]. Contrast-enhanced MRA rapidly acquires T1-weighted images during bolus administration of gadolinium-based intravenous contrast media of 0.1 to 0.2 mmol/kg (maximum dose: 0.3 mmol/kg) [48]. In a routine protocol, images are generally acquired using fast spoiled gradient recalled echo-based sequences (FSPGR) [48]. The most important parameter is the optimal timing of acquisition, since intracranial veins may be enhanced and interfere with the arteries, if timing of acquisition is not accurate, thus reducing the quality of the MRA images. Various different techniques have been introduced for optimal timing as time estimate, bolus test, or automatic triggering. Contrast-enhanced (CE) MRA combined with postprocessing techniques requires approximately 10 to 40 sec acquisition time [49]. Even though this method has many advantages for imaging the body and the extracranial blood vessels, it is not widely used for imaging intracranial vessels.

The preferred method of imaging intracranial aneurysms with MRA is the 3D TOF technique, since it provides high-quality images, without contrast administration. It has better resolution and signal-to-noise ratio (SNR) and requires less time than PC MRA [50, 51]. However, this method is prone to artifacts produced by turbulent blood flow. Turbulent flow is most commonly observed at the carotid siphon and in large-size aneurysms. Performing 3D TOF reduces such artifact with the application of short TE. Nevertheless, 3D TOF sequence acquires a large slab with resulting signal loss, which may reduce signal intensity within the aneurysm, and thus may underestimate the size of the aneurysm. Moreover, turbulent flow artifact at the base of the skull, in combination with susceptibility artifacts, may exacerbate this phenomenon, thus decreasing the sensitivity of this method in depicting aneurysms at the skull base [50, 52, 53]. The introduction of MIP reconstruction, flow compensation, application of short TE, and smaller slice thickness may eliminate these artifacts [54].

Giant or thrombosed aneurysms presenting slow flow may also be poorly visualized with 3D TOF. These aneurysms will be better identified on the axial Spin Echo MR images in combination with MRA. Contrast-enhanced MRA eliminates the problem of thrombosed and giant aneurysms, since the injected contrast will fill the aneurysmal lumen [50, 55, 56]. However, the presence of SAH may lead to false interpretation due to the presence of increased signal intensity of blood products, which could be superimposed [57]. Improved imaging techniques and higher magnetic fields have been introduced in an effort to reduce such artifacts, reduce scan time, optimize SNR, and increase sensitivity and specificity, in order to establish this diagnostic modality as a screening tool.

## 4. MRA Performed at 3 Tesla

The introduction of 3 T into clinical practice provides increased signal to noise ratio (SNR), which is almost double compared to 1.5 T [58]. 3 T MRI permits very short repetition and echo times, making possible shorter acquisition times and larger field coverage, with improved spatial resolution compared to 1.5 T [53, 59, 60]. Moreover, imaging at 3 T provides superior spatial resolution, thus improved vessel contrast, better background tissues, and fat suppression, providing superior image quality and better visualization of intracranial vessels [50, 61, 62]. Willinek et al. compared 3 T with 1.5 T 3D TOF MRA and concluded that 3 T showed improved spatial resolution and better evaluation of the peripheral segments of intracranial vessels [63]. The increased SNR at 3 T results in MRA sequences with either shorter scan times and unchanged resolution compared to lower magnetic fields or increased resolution at the same scan time. MRA at 3 T reportedly permits 30% higher SNR and 15% higher contrast to noise ratio (CNR) compared to 1.5 T [62–64]. Gibbs et al. reported that 3D TOF MRA at 3 T had more clear depiction of intracranial aneurysms compared to 1.5 T, even though all aneurysms were detected on 1.5 T [61]. In a study performed by Nowinski et al. [65], comparing imaging of blood vessels at 1.5 T, 3 T, and 7 T, they concluded that for imaging of arteries, 3 T is better than 1.5 T and 7 T, while, for vein imaging, 7 T is better than 1.5 T and 3 T [65]. Regarding the morphology of the aneurysm, the application of newer postprocessing techniques and the introduction of volume rendering, have contributed in excellent identification of the shape of the aneurysm [66]. This increase of SNR may allow voxel diameters as low as 1 mm^3^, resulting in much better identification of vessel contour and subsequently better delineation of the aneurysm morphology. Even though the location of the aneurysm is clearly depicted, its morphology depends on the applied imaging protocol, which requires high SNR and good suppression of stationary tissues. Moreover, the morphology of the aneurysm as well as its relationship with the parent vessel has increased with the application of 3 T and newer postprocessing techniques. The neck of the aneurysm is also clearly depicted with the application of background suppression methods and the increased SNR at 3 T.

Application of special techniques improves surrounding tissue suppression with the drawback of increasing scan time [67–69]. Magnetization transfer (MT) technique separates macromolecules from water, thus providing better suppression of the background tissues and better depiction of the vessels, maintaining meanwhile a high SNR. However, MT requires larger acquisition times and, at 3 T, may increase specific absorption rates (SAR), causing increased heating of the tissues [69–71]. Therefore, all 3 T MRI scanners have an automatic protection mechanism, which is activated when safe SAR limits are exceeded. Chemical shift-based fat suppression technique is suppressing high signal of fat, which may be misinterpreted as vascular pathology [68]. The application of this technique is particularly helpful for imaging vascular structures at the base of the skull. Both of these techniques (MT and chemical shift fat saturation) may increase scan time, which may be reduced by applying parallel imaging.

**Figure 1:**
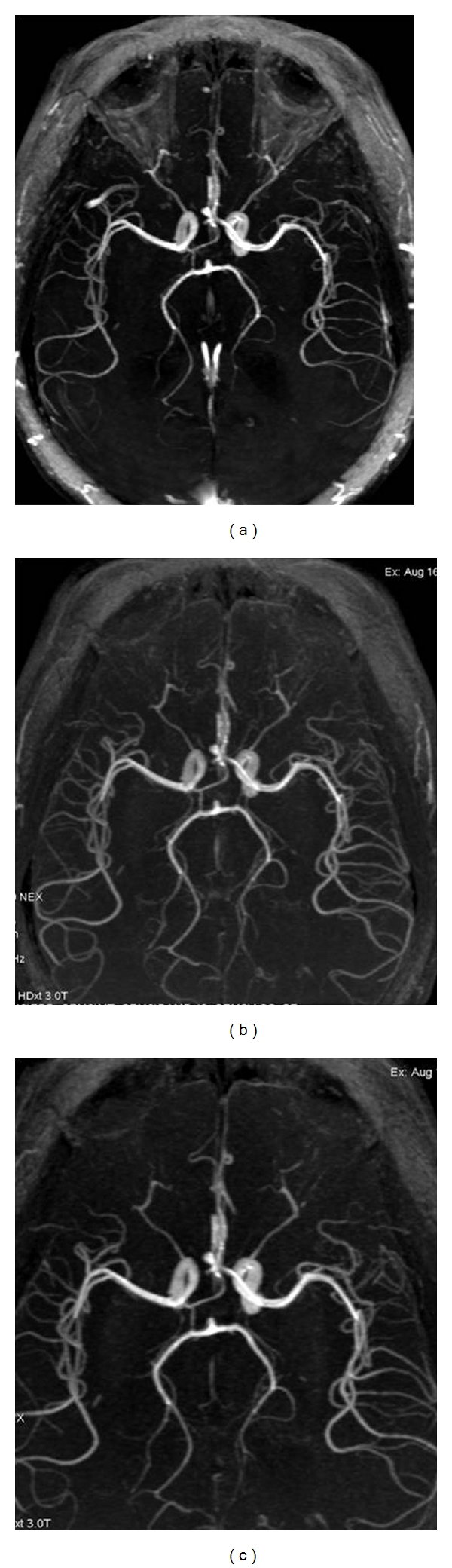
(a) 3D TOF MRA at 3 T at the level of the circle of Willis performed without MT or FS and without application of parallel imaging (ASSET). The scan time was 4 minutes and 53 seconds. Intraorbital fat signal is not completely compressed and is superimposed on the images, obscuring the visualization of the ophthalmic arteries. (b) 3D TOF MRA at 3 T at the level of the circle of Willis performed without MT but with FS and with the application of parallel imaging (ASSET). The scan time was 2 minutes and 34 seconds. Intraorbital fat signal is completely compressed, and only the arteries are clearly visualized. (c) 3D TOF MRA at 3 T at the level of the circle of Willis performed with MT, with FS, and with the application of parallel imaging (ASSET). The scan time was 2:03 min. The arteries are much clearly identified. All images clearly show the Acom aneurysm, but images with application of FS and MT are much clearer and required the shorter scan time.

**Figure 2:**
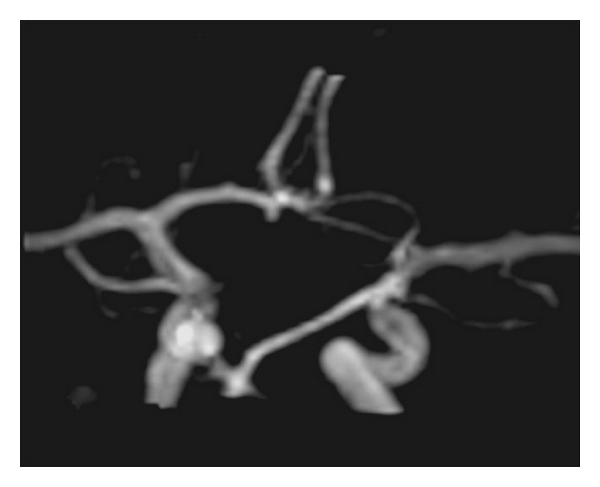
3D TOF MRA at the level of the circle of Willis performed at 3 T with application of MT and FS clearly shows a very small (2 mm) aneurysm at the A1 segment. This finding was confirmed with the DSA.

Parallel imaging is a technique, which uses information from multiple-channel receive coils to replace some phase encoding steps, allowing reduction of acquisition time [72] (Figure 1). The limitation of this technique is slight reduction of the SNR. However, since SNR is very high at 3 T, parallel imaging is routinely applied resulting in reduced scan times, with adequate image resolution. Parallel imaging makes submillimeter voxel dimensions possible, resulting in significant improvement of vessel identification and better delineation of vascular morphology. It has been postulated that MRA at 3 T permits visualization of intracranial aneurysms, as small as 1 mm [50, 61, 68, 73] (Figure 2). The application of parallel imaging and multichannel coils at 3 T results in increased SNR and reduced scan time, allowing optimal background suppression and providing better image quality [30, 66, 72, 74].

As above mentioned, 3D TOF MRA may miss aneurysms with slow or turbulent flow. Contrast-enhanced MRA provides better depiction of this aneurysm, being less prone to signal intensity losses due to turbulence or flow saturation. However, it is more invasive, requiring an ultrafast, bolus injection of intravenous contrast media [55]. Contrast enhanced MRA was also improved from high-field MRI at 3 T, due to improved spatial and temporal resolution. Increased gadolinium effect at 3 T can also result in reduced contrast volume, is easier to be performed, and may cover larger areas extending from the aortic arch to the intracranial vessels, simultaneously [56, 62, 66, 73, 74]. Additional imaging of giant or slow-flow aneurysms may be performed with PC MRA [50]. Moreover, evaluation of SE T1-weighted images nicely delineates thrombosed aneurysms and may provide complementary information regarding the size of the thrombus.

It is generally accepted that meticulous knowledge of the flow dynamic characteristics of an aneurysm is of paramount importance for assessing the possibility of rupture in cases of unruptured aneurysms and also for the treatment planning, either microsurgical or endovascular [75, 76]. Adequate depiction of the regional blood flow and its dynamics becomes essential for evaluating intracranial aneurysms and also for minimizing the chance of aneurysmal recanalization in cases of endovascular treatment [75, 77]. Phase-contrast MRI is the method of choice since it can depict dynamics of flow in the vessels. Phase-contrast MRA may theoretically measure flow velocity at the neck of an aneurysm. Hollnagel et al. [78] measured flow velocity in a mechanical experimental model by 3D PC MRA and concluded that it may identify the velocity profile of an aneurysm. Similarly, Yamashita et al. reported that, by implementing a 4D flow technique, they were also able to assess *in vivo* 3D hemodynamics of intracranial vessels, making this a promising method as well [79]. However, it has to be emphasized that these methods are investigational and require further evaluation and validation before being implemented into the clinical practice.

Additionally, 3D TOF MRA has been used in following up the patients with coiled aneurysms. Majoie et al. in a study comparing 3 T TOF, CE-MRA, and DSA reported 81% agreement between the employed methods [80]. In 14% of their cases, rim artifacts from the presence of the implanted coils did not interfere with the interpretation of the occlusion status of the studied aneurysms. Therefore, they concluded that 3 T TOF MRA and CE-MRA are a promising method in the evaluation of residual flow in a treated aneurysm [80].

## 5. Limitations

Imaging at 3 T presents several limitations and drawbacks generated by the prolonged acquisition time and the high magnetic field. The incidence of susceptibility artifacts at 3 T is double of that at 1.5 T. The occurrence of susceptibility artifacts appears to be higher at the skull base and near the bone-air interphase. Furthermore, the presence of metallic implants produces signal loss and image distortion. It has also been reported that increased susceptibility artifacts occur in the presence of intravascular coils [80]. However, it has been reported that these artifacts have minimal effect on MRA's diagnostic capacity [80].

MRA may be influenced by motion artifacts, due to the long acquisition time, making the evaluation of intubated and noncooperative patients quite challenging. This effect is exacerbated if a long field of view needs to be covered.

Turbulent flow may also result in signal loss, thus degrading image quality, particularly in large-sized aneurysms. Shortening the TE may minimize this artifact. Incomplete suppression of stationary tissue or venous flow may interfere with the blood vessels, thus degrading image quality.

**Figure 3:**
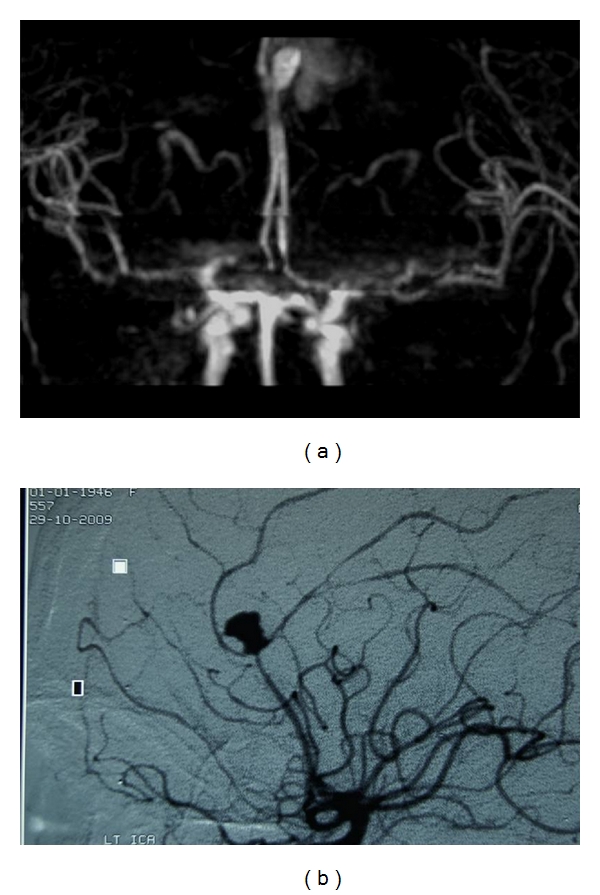
(a) 3D TOF MRA performed at 3 T clearly shows the pericallosal aneurysm. However, MRA also shows the surrounding hemorrhage, which slightly obscured the images, even though the aneurysm is clearly depicted. (b) DSA clearly identifies the aneurysm without superimposed hemorrhage.

Small-sized aneurysms (<3 mm) may not be clearly identified on MRA, particularly if they are located near the skull base or if they are obscured by motion artifacts. Moreover, structures with increased signal on T1-weighted image as thrombus, slow flow, blood products, or fat may be misinterpreted as an aneurysm (Figure 3). Contrast-enhanced MRA may eliminate these artifacts. However, it is also influenced by the increased signal of stationary tissues on T1-weighted image; it depends on optimal timing of contrast injection and may present allergic reactions due to the injected contrast. Moreover, CE-MRA may not be performed in patients with renal failure due to the increased risk of nephrogenic systemic fibrosis.

## 6. Conclusions

Magnetic resonance angiography constitutes a very sensitive and accurate noninvasive imaging modality, for evaluating patients with suspected, nonruptured, and intracranial aneurysms. It is applicable to cooperative patients, and it can detect even small-sized aneurysms (<3 mm), delineating their exact shape, their size, and their relationship to the adjacent vessels and can even provide valuable information regarding their hemodynamic characteristics. Furthermore, MRA can be applied in the followup of patients treated with endovascular coiling. Various MRA techniques have been developed; however, the 3D TOF seems to be the most sensitive and accurate one. In special occasions, this may be complemented by the performance of CE MRA. The application of 3 T MRA further improves MRA's sensitivity by increasing the SNR and the spatial resolution, while minimizing the examination time. Limitations such as susceptibility and motion artifacts need to be taken into consideration for avoiding misinterpretation of MRA findings. Newer MRA methodologies and further increase of the magnetic field strength may further improve MRA's sensitivity and minimize its limitations.
